# Metagenomes, metagenome-assembled genomes, and metatranscriptomes from a chlorinated ethene-dechlorinating culture amended with biochar pyrolyzed at different temperatures

**DOI:** 10.1128/mra.00104-24

**Published:** 2024-08-07

**Authors:** Hongyu Dang, Weilun Zhao, Timothy E. Mattes

**Affiliations:** 1 Department of Civil and Environmental Engineering, University of Iowa, Iowa City, Iowa, USA; University of Notre Dame, Notre Dame, Indiana, USA

**Keywords:** *Dehalococcoides*, chlorinated ethenes, bioaugmentation, biochar, methanogens

## Abstract

We investigated the effects of biochar and pyrolysis temperature on a chlorinated ethene-dechlorinating anaerobic consortium. Sequencing of nucleic acids from suspended and biochar-attached cells yielded 9 metagenomes, 122 metagenome-assembled genomes, and 18 metatranscriptomes that provide insights into the structure, function, activity, and interactions of the dehalogenating consortium with biochar.

## ANNOUNCEMENT

Biochar has been used as a supplement to enhance anaerobic biodegradation of chlorinated ethenes, as it provides habitat, facilitates electron transfer, and stimulates microbial activity and microbe-microbe interactions (1, 2). However, specific biochar properties (e.g., pore size, electron donating/accepting, conductivity, etc.) that are beneficial to microbial ecology, growth, and activity are poorly understood. To fill these research gaps, biochars were synthesized at various temperatures (350°C, 500°C, 700°C, and 900°C) to provide a range of material properties. Each biochar type was added to duplicate microcosms constructed with 100 mL RAMM medium in 160 mL serum bottles and inoculated with an anaerobic tetrachloroethene-dechlorinating consortium (SDC-9) (3–5), together with duplicate live controls without biochar (10 bottles in total).

After 51–82 days of incubation, solid biochar was separated from the liquid culture in bottles by passing through 20 µm UV-sterilized filters (Just the Basics, Woonsocket, RI, USA) and washed with RAMM medium. RNA and DNA were extracted from both liquid and biochar samples with the RNeasy PowerSoil total RNA kit plus DNA Elution kit (Qiagen, Germantown, MD, USA). Residual DNA in RNA extracts was removed with the TURBO DNA-free kit (Thermo Fisher Scientific) and the Direct-zol RNA MiniPrep Plus kit (Zymo Research Corp., Irvine, CA, USA). High-quality RNA (i.e., with clear 16S and 23S rRNA peaks and RNA integrity number > 8), as confirmed with a 2100 Bioanalyzer RNA Pico assay (Agilent Technologies, Santa Clara, CA, USA), was submitted for sequencing.

RNA and DNA were sequenced at the Iowa Institute of Human Genetics (Iowa City, IA, USA). Sheared DNA (average size 550 bp) was used to prepare indexed DNA libraries with the KAPA HyperPrep Kit (Roche Sequencing and Life Science, Indianapolis, IN, USA). RNA libraries were indexed and rRNA-depleted with the stranded total RNA preparation with Ribo-Zero Plus Kit (Illumina Inc., San Diego, CA, USA). Supplemental rRNA probes (149) for dominant taxa in the culture, developed from eight 16S rRNA sequences retrieved from Silva 138.1 (6), were included to improve rRNA depletion and mRNA sequencing efficiency. The DNA and RNA libraries were pooled and sequenced with NovaSeq 6000 on SP (150-bp paired-end) and S1 flow cells (100-bp paired-end), respectively.

**Fig 1:**
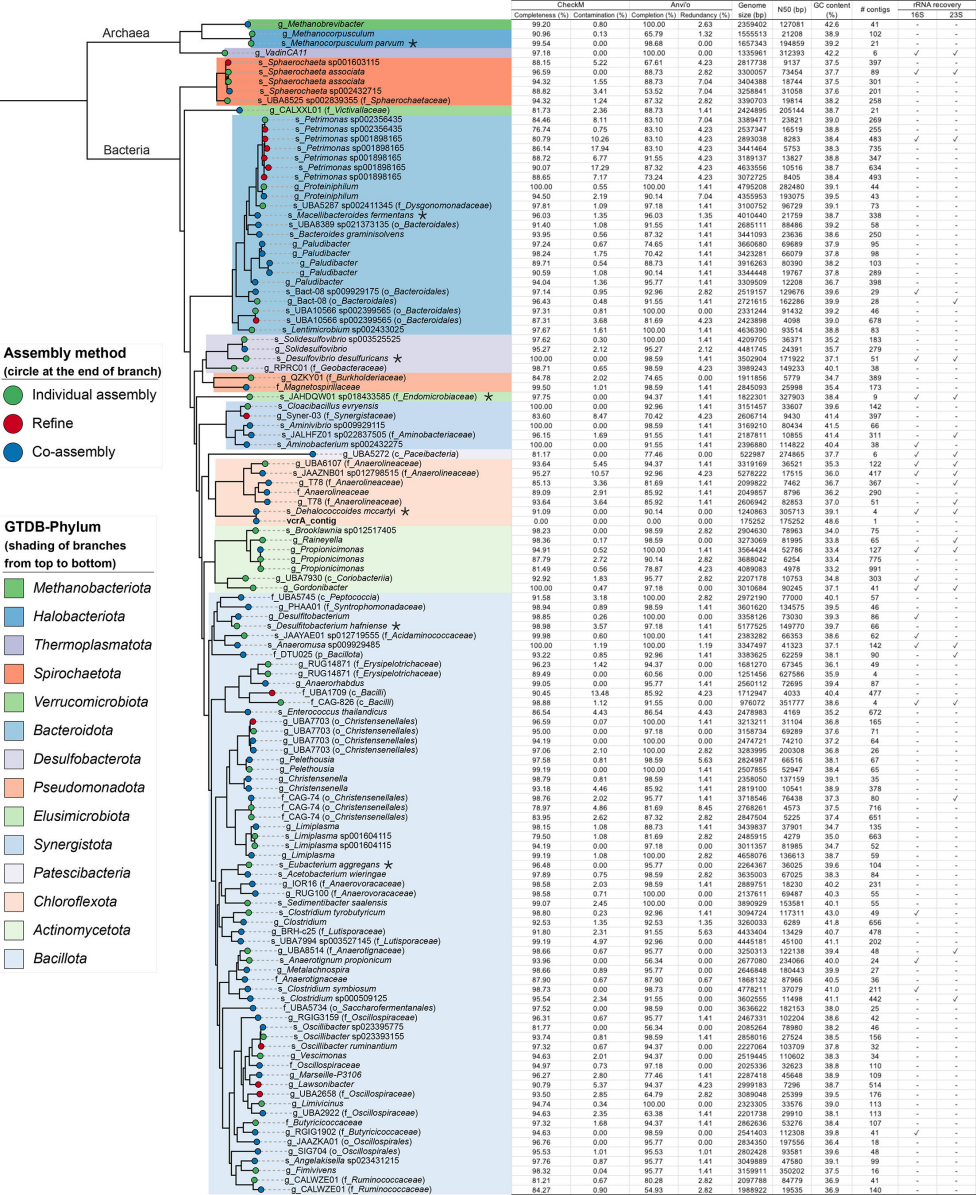
Phylogenetic tree of 122 MAGs after dereplication, quality statistics determined by CheckM and Anvi’o, genome size, *N*_50_, GC content, number of contigs, and rRNA gene recovery (i.e., 16S and 23S, “✓” represents a recovery and “-” represents no recovery). Anvi’o was used to refine bins with completion ≥ 80% and contamination > 5%. Redundant sequences were manually removed based on their read coverage according to bin refinement instructions (https://merenlab.org/2015/05/11/anvi-refine/). The tree was generated using marker genes from each MAG as determined by checkM. The contig containing *vcrA* (vcrA_contig) was manually added next to the *Dehalococcoides* MAG on the tree as it has a 99% nucleotide identity with *Dehalococcoides mccartyi* determined by BLAST (19). Because the vcrA_contig and *Dehalococcoides* MAG had different tetranucleotide frequencies, they were binned separately. The vcrA_contig GenBank accession number is PP061217.1. The 16S rRNA gene sequences of dominant taxa (marked with “∗”) were collected from the Silva database to design probes for rRNA depletion. Silva taxa (and accession numbers) are *Eubacterium* (QTVG01000004); *Dehalococcides mccartyi*, (CP011127); *Desulfitobacterium hafniense* (AP008230); *Desulfovibrio biadhensis* (LM999902); *Methanocorpusculum aggregans* (LMVO01000026); *Endomicrobium proavitum* (CP009498); and *Macellibacteroides fermentans*, a(HQ020488).

Raw sequencing reads were quality controlled by trimming adapters and low-quality sequences (average quality score per base < 15 or sequence length < 36 bases) with Trimmomatic (version 0.39) (7). Trimmed DNA and RNA data sets ranged from 38 to 60 million reads (Table 1), which provided sufficient sequencing depth for metagenomic and metatranscriptomic analysis (8). Reads were assembled into contigs using both individual assembly and co-assembly approaches with Megahit (version 1.2.9) (9). Trimmed short reads were mapped to the contigs with bowtie2 (version 2.2.5) (10). Contigs and their corresponding BAM files were used for binning with Metabat2 (version 2.15) (11). Bin completion and contamination were quantified with CheckM (version 1.2.2) (12) and Anvi’o (version 7.1) (13). Bins with a completion ≥ 80% and contamination ≤ 5% were designated as metagenome-assembled genomes (MAGs). Bins with a completion ≥ 80% and contamination > 5% were further refined with Anvi’o. All MAGs were dereplicated with dRep (version 3.4.2) (14). MAG marker genes, determined by checkM, were aligned with Clustal Omega (version 1.2.3) (15) and used to generate a phylogenetic tree with iqtree (version 2.0.3) (16). MAG taxonomy was classified using GTDB-tk (version 2.1.1; database release R214) and visualized in iTOL (version 5) (17) (Fig. 1). MAGs were annotated using the NCBI Prokaryotic Genome Annotation Pipeline (version 6.0) (18).

**TABLE 1:** Accession numbers and characteristics before and after trim of metagenomes and metatranscriptome short reads^*a*^

Sample name	SRA accession no.	BioSample no.	Pyrolysis temperature (°C)	Replicate	Sample type	Number of reads			
						Before trim	After trim-paired	After trim-unpaired R1	After trim-unpaired R2
Char350-A-liquid_DNA	SRR27243496	SAMN38883632	350	–^*b*^	Metagenome	42,164,008	40,942,698	841,272	287,877
Char350-B-attached_DNA	SRR27243495	SAMN38883633	350	–	Metagenome	53,558,356	52,003,807	1,030,067	386,197
Char500-A-attached_DNA	SRR27243484	SAMN38883634	500	–	Metagenome	44,813,995	43,558,176	816,976	326,457
Char500-B-liquid_DNA	SRR27243476	SAMN38883635	500	–	Metagenome	47,600,200	46,070,842	1,020,842	364,793
Char700-A-attached_DNA	SRR27243475	SAMN38883636	700	–	Metagenome	49,407,025	48,054,961	848,396	379,281
Char700-B-liquid_DNA	SRR27243474	SAMN38883637	700	–	Metagenome	50,301,937	48,829,200	952,342	385,050
Char900-A-liquid_DNA	SRR27243473	SAMN38883638	900	–	Metagenome	48,444,322	47,201,060	794,642	338,191
Char900-B-attached_DNA	SRR27243472	SAMN38883639	900	–	Metagenome	48,296,394	46,942,738	849,928	377,311
NoChar-A-liquid_DNA	SRR27243471	SAMN38883640	NA^*c*^	–	Metagenome	46,581,840	45,244,312	833,038	378,801
Char350-A-attached_RNA	SRR27243470	SAMN38883641	350	A	Metatranscriptome	57,957,767	56,961,929	652,912	284,891
Char350-A-liquid_RNA	SRR27243494	SAMN38883642	350	A	Metatranscriptome	48,115,301	47,220,372	698,649	145,630
Char350-B-attached_RNA	SRR27243493	SAMN38883643	350	B	Metatranscriptome	48,588,053	47,579,440	796,578	143,771
Char350-B-liquid_RNA	SRR27243492	SAMN38883644	350	B	Metatranscriptome	51,770,161	50,826,589	710,920	165,394
Char500-A-attached_RNA	SRR27243491	SAMN38883645	500	A	Metatranscriptome	57,882,442	56,775,702	831,448	203,673
Char500-A-liquid_RNA	SRR27243490	SAMN38883646	500	A	Metatranscriptome	57,447,734	55,879,132	1,329,767	158,883
Char500-B-attached_RNA	SRR27243489	SAMN38883647	500	B	Metatranscriptome	54,685,915	53,406,752	1,054,073	147,996
Char500-B-liquid_RNA	SRR27243488	SAMN38883648	500	B	Metatranscriptome	53,516,674	52,587,866	702,192	159,253
Char700-A-attached_RNA	SRR27243487	SAMN38883649	700	A	Metatranscriptome	39,154,854	38,428,073	512,125	169,707
Char700-A-liquid_RNA	SRR27243486	SAMN38883650	700	A	Metatranscriptome	60,394,555	59,237,767	918,897	169,161
Char700-B-attached_RNA	SRR27243485	SAMN38883651	700	B	Metatranscriptome	66,681,063	65,239,485	1,150,431	196,916
Char700-B-liquid_RNA	SRR27243483	SAMN38883652	700	B	Metatranscriptome	58,947,262	57,787,395	921,054	166,568
Char900-A- attached_RNA	SRR27243482	SAMN38883653	900	A	Metatranscriptome	58,700,889	57,558,788	843,335	211,378
Char900-A-liquid_RNA	SRR27243481	SAMN38883654	900	A	Metatranscriptome	66,675,677	65,344,965	1,061,992	181,614
Char900-B-attached_RNA	SRR27243480	SAMN38883655	900	B	Metatranscriptome	62,066,578	60,842,217	969,950	178,548
Char900-B-liquid_RNA	SRR27243479	SAMN38883656	900	B	Metatranscriptome	54,717,891	53,611,439	873,365	160,818
NoChar-A-liquid_RNA	SRR27243478	SAMN38883657	NA	A	Metatranscriptome	51,288,938	50,335,534	743,633	146,313
NoChar-B-liquid_RNA	SRR27243477	SAMN38883658	NA	B	Metatranscriptome	60,643,367	59,652,887	685,617	235,730

^*a*^ In total, 9 (of 18) DNA samples and all 18 RNA samples were sequenced.

^*b*^ –, no replicate for this sample.

^*c*^ NA, biochar was not available for this sample.

A total of 122 MAGs belonging to 14 phyla were obtained, including one classified as *Dehalococcoides mccartyi* (Fig. 1). At least 87% of the metagenomic short reads were mapped to these MAGs, which indicated they represent the microbial composition in SDC-9. These data will advance knowledge of chlorinated ethene-dechlorinating microbial communities and their interactions with biochar.

## Data Availability

Raw sequence data and MAGs are available under NCBI BioProject number PRJNA1054096, with metagenome and metatranscriptome SRA accession numbers from SRR27243470 to SRR27243496.
